# Multicenter data acquisition made easy

**DOI:** 10.1186/1745-6215-11-49

**Published:** 2010-04-30

**Authors:** Jacob Rosenberg, Nadia Abdelaal Henriksen, Lars Nannestad Jørgensen

**Affiliations:** 1 Department of Surgery, Herlev Hospital, University of Copenhagen, Denmark; 2 Department of Surgery, Bispebjerg Hospital, University of Copenhagen, Denmark

## Abstract

**Background:**

The process for data collection in multicenter trials may be troublesome and expensive. We report our experience with the spreadsheet function in Googledocs for this purpose.

**Methods:**

In Googledocs the data manager creates a form similar to the paper case record form, which will function as a decentral data entry module. When the forms are submitted, they are presented in a spreadsheet in Googledocs, which can be exported to different standard spreadsheet formats.

**Results:**

For a multicenter randomized clinical trial with five different participating hospitals we created a decentral data entry module using the spreadsheet function in Googledocs. The study comprised 332 patients (clinicaltrials.gov identifier: NCT00815698) with five visits per patient. One person at each study site entered data from the original paper based case report forms which were kept at the study sites as originals. We did not experience any technical problems using the system.

**Conclusions:**

The system allowed for decentral data entry, and it was easy to use, safe, and free of charge. The spreadsheet function in Googledocs may potentially replace current expensive solutions for data acquisition in multicenter trials.

**Trial registration:**

clinicaltrials.gov NCT00815698

## Background

Data collection in multicenter studies may be troublesome because of considerable amounts of administrative paperwork or the necessity to adopt very expensive commercial solutions. It is normally required to photocopy paper based case record forms (CRFs) and send them to a coordinating centre implying a risk of data loss and considerable delay. Commercial solutions with scanning of CRFs are expensive and still require transportation of the paper based CRFs to the trial administration unit [1,2].

There is, however, now a simple, effective and free solution available. The present paper describes an asynchronous collaborative spreadsheet system that allows for decentral data entry using the spreadsheet function in Googledocs [3].

## Methods

In this section we are going to describe the necessary steps required to use Googledocs for data acquisition.

### Creating the master file

Initially, all data collectors must have a Googledocs or Gmail account. This is free of charge and very easy to establish. At the Googledocs website, press "Get started" and then get registered with your email and a password.

The research group nominates a data manager with universal access and permissions to the master file (see below). The data manager then creates a master file as a form in Googledocs. The design of the master file represents the standard paper based CRF which thereby defines the format of each data parameter. Later on, when the forms are submitted, they will be presented in a spreadsheet format in Googledocs.

**Figure 1:**
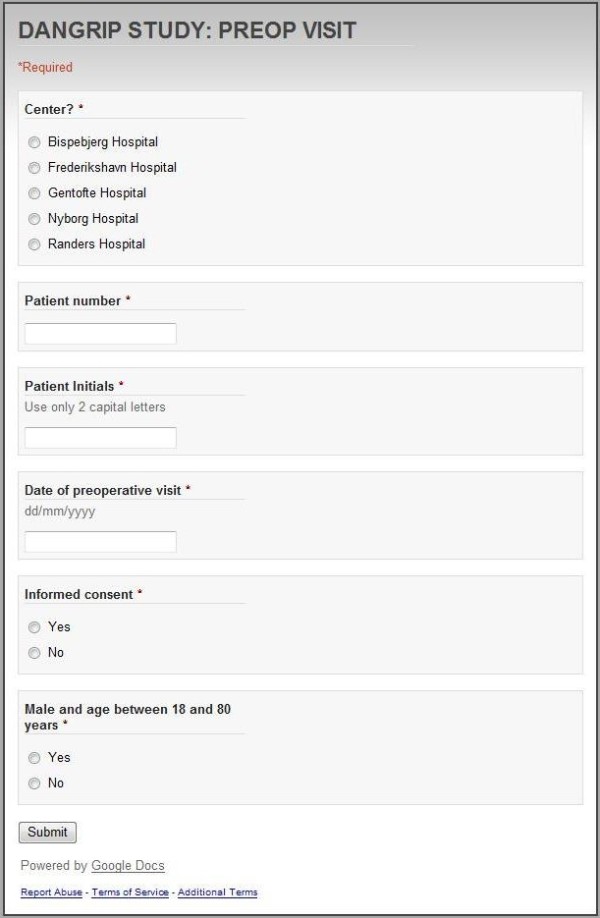
**Case record form as presented for the decentral data entry person when entering data**.

When entering Googledocs press "create new" - then choose "form". Thereafter, a new window provides you the opportunity to give the item a headline and choose between different question types, e.g. text, paragraph text, multiple choice, textboxes, choose from a list, scale and grid. After defining the first item, you will press "add item" and similar procedures are completed again until all items on the CRF have been defined in your Googledocs form (Figure 1).

For each item you can choose the option "required question" to obviate submission of the form before the completion of this item. If you create a long form, you can insert page breaks and give each page a title. In this way, you can make it possible for the data entry persons to skip items that might be irrelevant and direct them to items that must be answered. To do this, you add the item "multiple choice" and select the option "go to page based on answer". If you should experience any trouble creating the master file, the system provides a sound online manual to guide you through the setup process.

Each patient visit (e.g. preoperative, one week, one month etc.) may be represented by the creation of a unique database. This will give the data entry person the possibility to enter data after each patient visit. Thus, the central data manager will have the opportunity to closely follow the progression of the study. After study completion the different databases may be linked using standard Excel functions or similar.

### Defining roles for the data collectors

The central data manager has the opportunity to assign different roles for each decentral data entry person as defined by the various permission levels available in Googledocs. When creation of the form is finished, press "share" and "invite people" and then enter the email addresses of the data entry persons. When sending the invitations, the data manager can choose if the data entry persons should have permission to enter data, to view data and/or to edit data. If the data entry persons only have permissions to enter data at the decentral sites, then these persons will not be able to view or edit data afterwards, which will only be possible for the data manager. If the data manager gives the decentral data entry persons permission to enter and view data, then they can view the original data inside the database and compare these with the original CRFs. In this way, it is possible to perform a validation procedure of the data entered into the database.

The data manager invites the data entry persons using the "share" function by sending invitations to their e-mail addresses. After this, the data entry persons can log on using their username and password to Googledocs.

The system allows multiple users at different trial sites to actually enter data simultaneously. The data will appear in the master file immediately upon submission. Each data entry will get a time stamp in order for the data manager to keep track of data submissions.

### Finalizing the database

The submitted forms are presented in a spreadsheet in Googledocs, which can be exported to different standard spreadsheet formats, e.g. Microsoft Excel or similar. The data manager has the opportunity to create copies of individual databases during the data submission period for data backup or interim analyses.

When all data have been entered, the spreadsheet can be exported to another spreadsheet format, and the Googledocs database can be deleted thus finalizing the data entry period.

### Security

Like any other web-based practice management system the log-on procedure to Googledocs requires a username and password. As default, however, the transfer of data by Googledocs is not encrypted. Two steps are highly recommended because they greatly improve safety: 1) Type https://docs.google.com instead of http://docs.google.com. The extra "s" means a Hyper Text Transfer Protocol *Secure *meaning that the computers "agree" on a code between them, and they then encrypt the messages between them, significantly reducing the risk of a hacker to interpret the communication. The computers at each end use a document called a SSL certificate (128 bits that is considered strong) for the encryptation process. The only down-side effect of the encryptation is that the traffic is a little slower. 2) Do not check "remember my password" when logging in and do not forget to log-out of Googledocs when leaving the computer. There are also commercial packages available (e.g. DocCloak for Groups) that work as browser add ons and provide high level encryptation.

### Technical compatibility

Googledocs will run on all current major operating systems and browsers: Windows XP/NT/Vista (Internet Explorer 7 or 8, Firefox 3.0, Google Chrome), Linux Ubuntu (Firefox 3.0) and Mac OSX (Safari 3 or 4, Firefox 3.0). No matter what browser is used one has to *enable cookies *and *enable JavaScript*.

## Results

We have used the spreadsheet function in Googledocs for decentral data entry into a central database for a multicenter randomized clinical study, conducted at five different hospitals and including 332 patients (clinicaltrials.gov identifier: NCT00815698). The study comprised five patient visits: preoperative, intraoperative, one month, 12 months and end of study. Before entering real patient data we made a test module where all decentral data entry persons entered fictive patient data. This gave the involved persons experience using the data-entry-form and allowed identification of logistical problems in the design of the database. After this test period, the involved data entry persons as well as the data manager did not experience any problems what so ever using the system.

Even though we encountered no technical problems using the system, there have been various uncertainties with different data item definitions etc. In this case it has been a good experience to have a dedicated key person to whom these questions could be addressed. In our case, this has been the data manager who could answer questions by e-mail on a day-to-day basis.

The original paper based CRFs were kept at the decentral centres and these CRFs had the original patient identification information such as social security numbers. In the Googledocs database the social security number was not entered so that the patients' anonymity was kept according to our national law regarding data safety. The original paper based CRFs are kept at the various trial centres for 15 years, again according to our national laws.

## Discussions

We have demonstrated a suitable tool for data acquisition in multicenter trials with the use of the spreadsheet function in Googledocs. This system is simple to use, free and safe. Furthermore, it is possible to tightly follow the speed of patient inclusion. The laws regarding data protection are easily followed with separation of patient identification information from the central database containing data from all participating centres. Thus, the final data file does not contain person-sensitive data. Furthermore, the workload for the coordinating centre is minimized since data entry is performed by every single participating centre.

Other groups have used Googledocs as a tool for research purposes. This has to our knowledge only been reported for writing research protocols [4,5]. These groups have used the text function in Googledocs for collaborative writing. Another possibility within the Googledocs platform is the presentation module allowing both asynchronous editing of presentations and a simultaneous presentation for an audience placed at multiple locations [5]. Finally, Googledocs provides an opportunity for simultaneous online discussion between participating centres using the chat-function of the system [5].

Other internet-based systems for collaborative communication are available within e.g. pathology [6], chemistry [7], infectious diseases [8] and immunology [9]. We do not have any experiences with these fixed systems where the format is already defined. They may therefore not be suitable for data acquisition in multicenter trials.

Data safety is ensured by the use of passwords for all participating researchers. There is, however, a theoretical possibility for a computer hacker to view and change data [10]. It is therefore vital that the original CRFs are kept at each participating centre and it is recommended to perform a final data validation procedure at the end of all entries and before data analyses. As pointed out it is highly recommended to encrypt the data transfer to keep out anyone in between the sender and the recipient to open the message. Google's privacy policies state that 1) their products reflect strong privacy standards and services, 2) the collection of personal information is transparent and 3) Google is a responsible steward of the information they hold. When designing a multicenter trial the local Human Research Ethics Committee and Data Protection Agency must decide whether it is acceptable that databases *without patient identification information *are "exported" to a Google server located in another country. Within the Googledocs system it is possible to make regular backups of the original database files. The backup files can then be kept on other media according to local data protection laws.

## Conclusions

The use of the spreadsheet function in Googledocs for data acquisition in multicenter trials is an easy tool for all researchers around the world with an internet connection. Design of the data entry forms is easy and quick, and the design of each data entry form can be tailored according to the individual trial. We have used this system with success and have not encountered any technical problems during the process. Finally, the system is free of charge.

## Competing interests

The authors declare that they have no competing interests.

## Authors' contributions

All authors participated in the literature search, and the writing of the paper was done in close collaboration between all authors. All authors read and approved the final manuscript.
